# Predicting the expansion of *Gephyraulus lycantha* as a key pest of goji berry in China under climate change

**DOI:** 10.3389/fpls.2026.1786710

**Published:** 2026-04-10

**Authors:** Zhongkang Song, Jianling Li, Changrong Deng, Guozhen Duan, Guanghui Fan

**Affiliations:** Qinghai Plateau Tree Genetics and Breeding Laboratory, Laboratory for Research and Utilization of Qinghai Tibet Plateau Germplasm Resources, Academy of Agriculture and Forestry Sciences, Qinghai University, Xining, China

**Keywords:** gall midge, goji berry, maxent, distribution, climate change

## Abstract

**Background:**

The gall midge, *Gephyraulus lycantha* Jiao & Kolesik (Diptera: Cecidomyiidae), causes abnormal enlargement of goji berry, *Lycium barbarum* L, buds during its larval stage, forming galls and resulting in a significantly decrease in yield in China. Identifying the distribution of the midge in China under current and future climate change scenarios will provide guidance for the scientific prevention and control of this pest.

**Methods:**

The MaxEnt model was used to predict the current and future potential suitable habitats for the midge in China based on the filtered 56 distribution points and 11 environmental factors, and the ArcGIS software was used to analyze the changes in its suitable region.

**Results:**

The results showed that when the parameters were feature combination (FC) = HP and regularization multiplier (RM) = 1, the MaxEnt model was optimal, and the AUC and TSS values were greater than 0.90. The mean temperature of driest quarter (suitable range was -9.36–4.43 °C) was the most critical factor influencing the distribution of the midge. Under current climate conditions, the area of suitable habitat for the midge was 112.73 × 10^4^ km^2^, primarily distributed in Xinjiang (29.03 × 10^4^ km^2^), Inner Mongolia (26.44 × 10^4^ km^2^), Gansu (18.36 × 10^4^ km^2^), Qinghai (10.46 × 10^4^ km^2^), and Ningxia (3.90 × 10^4^ km^2^) Provinces. Under the 2050s and 2070s climate scenarios, the area of suitable habitats was larger than current ones (except for SSP126), reaching its maximum under the SSP585 (119.06 × 10^4^ km^2^) and SSP245 scenarios (135.25 × 10^4^ km^2^), respectively.

**Conclusion:**

In addition, climate warming would cause the suitable habitat of the midge to expand northeastward. Therefore, it is necessary to strengthen monitoring, early warning, and control measures for the pest to ensure the production of goji berry.

## 1 Introduction

**Figure 1:**
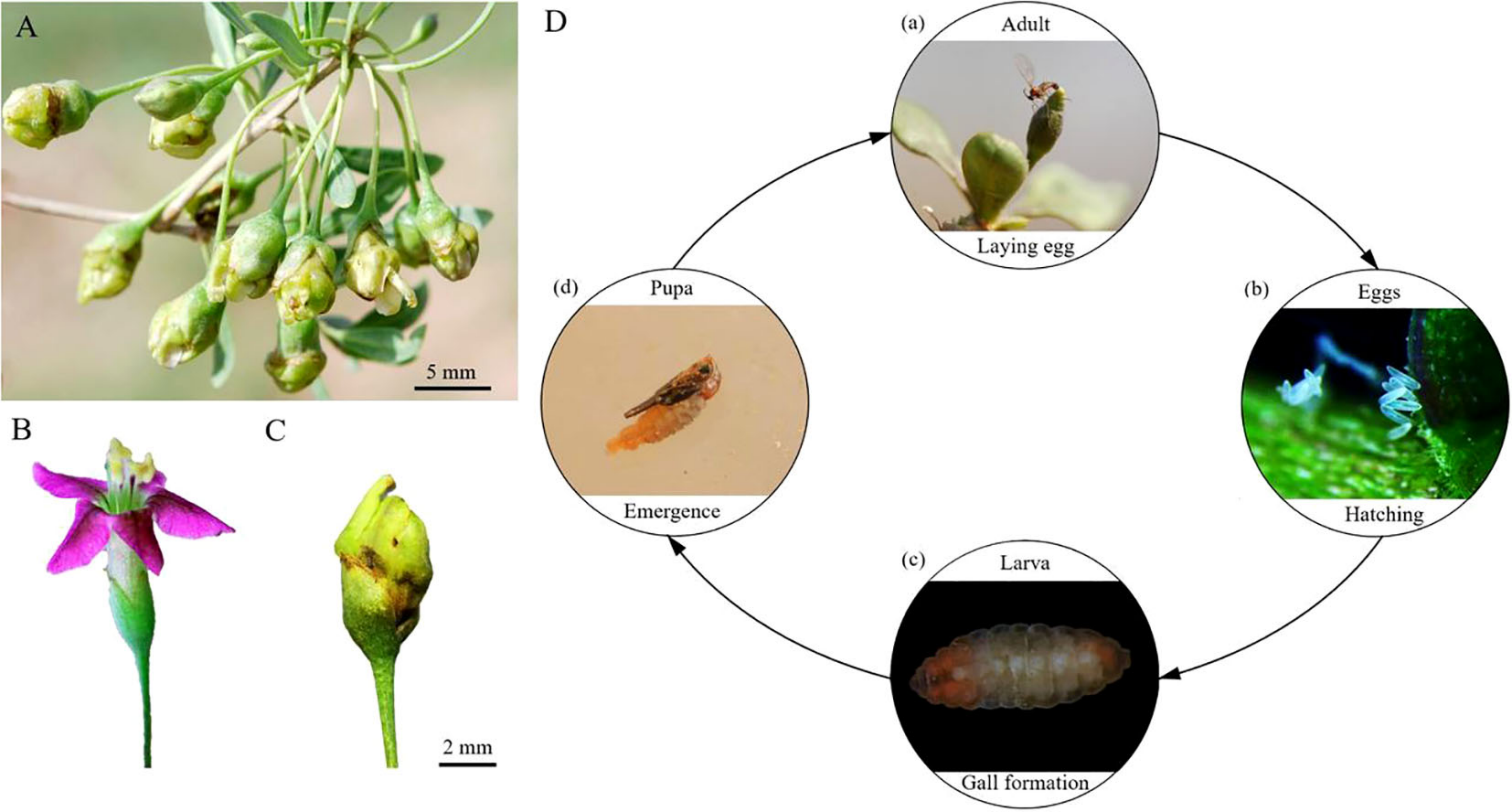
**(A)** The flower buds parasitized by *G. lycantha*; **(B)** Normal flower buds; **(C)** Parasitized flower buds; **(D)** The life history of *G. lycantha*. Photographs by Jianling Li and Haili Qiao.

*Lycium barbarum* L., commonly known as the goji berry, is a medicinal and edible herb indigenous to China. It has the effects of nourishing the liver and kidney, benefiting essence, and improving eyesight (Ma et al., 2023). Currently, the annual demand for goji berry is nearly 300,000 tons, with a cultivation area exceeding 1,200 km^2^ (Li et al., 2015). The entire industrial chain generates an income of over 32 billion RMB, making it a vital economic source for farmers and herdsmen in Northwest China (Song et al., 2024). However, goji berry undergoes simultaneous vegetative and reproductive growth with luxuriant branches and leaves, making it more susceptible to pests compared with other medicinal plants. The gall midge, *Gephyraulus lycantha* Jiao & Kolesik (Diptera: Cecidomyiidae), is a severe gall-forming pest that inflictes devastating damage on goji berry production (Zhang et al., 2023). Adults of the midge oviposit in the young buds of goji berry, and the hatched larvae bore into and feed on the ovaries (**Figures 1A-C**). This induces ovarian hypertrophy leading to gall formation, which inhibits the affected flower buds from normal anthesis and fructification. Mature larvae then burrow into the soil to pupate, completing the life cycle (**Figure 1D**) (Li et al., 2015). Due to the concealed nature of the larvae within the flower bud, the control efficacy of conventional pesticides, such as Deltamethrin and Imidacloprid, was poor because of their limited systemic absorption (Li et al., 2017). In recent years, with the continuous expansion of goji berry cultivation area, the distribution and damage range of the midge have increased annually. In severe cases, the infestation rate of the midge exceeds 60%, and the annual yield loss can reach 45%–65% (Wu and Li, 2014; Liu et al., 2020).

Over the past century, rising greenhouse gas emissions have driven significant global climate warming, with its impacts on ecosystems and the natural environment becoming increasingly pronounced (Pawan, 2014). As ectothermic animals, insects lack the ability to regulate their own body temperature, and their vital activities depend entirely on environmental temperature, making them highly sensitive to changes in ambient temperature (Bai et al., 2022). To cope with variations in environmental temperature, some pests can avoid unfavorable conditions through diapause regulation and seasonal migration (Peng et al., 2023; Zhang et al., 2025), whereas most insects adapt to climate change by modulating their internal metabolism (Durak et al., 2021). Within suitable temperature ranges, the growth and development of the midge are significantly promoted, while temperature fluctuations exert an impact on its life cycle. Changes in temperature regimes not only affect the growth, development, reproduction, and survival of the pests, but may also alter their population sizes and geographical distributions, and promote their expansion to higher latitudes and elevations (Nedvěd, 2009; Colinet et al., 2015). Therefore, studying the distribution of suitable habitats of the midge in China under climate change can provide a scientific basis for the prevention and control of this pest.

In the context of global climate change, Species Distribution Models (SDMs) are mathematical models based on niche theory, widely applied in pest prevention and monitoring, protection of rare species, biodiversity research, and assessments of the potential impact of climate change on species distribution (Santana et al., 2019; Rathore and Sharma, 2023). Among the available SDMs, the maximum entropy model (MaxEnt) has been used extensively both regionally and internationally in many fields owing to its the advantages of accurate prediction, simple operation, and high efficiency (Elith et al., 2006). This model comprehensively analyzes the distribution data of species and environmental factors to simulate suitable habitats in different regions (Sillero, 2011). In recent years, the model has been widely applied to predict the geographical distribution of the goji fruit fly *Neoceratitis asiatica* Becker (Song et al., 2024), the leaf beetle *Lema decempunctata* Gebler (Li et al., 2025b), *Aphis* sp (Ma et al., 2019), and their host plant *L. barbarum* (Wang et al., 2023).

Currently, the damage caused by the midge to its host plant is becoming increasingly severe. However, existing research on this pest has mainly focused on its biological characteristics (Zhang et al., 2021), behavior (Sun et al., 2023), and control strategies (Zhang et al., 2019). Studies on the geographical distribution and changes in suitable habitats of the midge under future climate scenarios remain unreported. The main objectives of this study are to: (1) identify the key environmental factors influencing its geographical distribution; (2) predict the distribution of potentially suitable areas for the midge in China under current climate conditions; and (3) forecast and compare the potentially suitable areas for the midge and the changing trends under different future (2050s, 2070s) climate scenarios (SSP126, SSP245, SSP370, SSP585). The findings will provide a theoretical basis for the monitoring and forecasting of the midge and guide the site selection of organic goji berry bases.

## 2 Materials and methods

### 2.1 Study area

**Figure 2:**
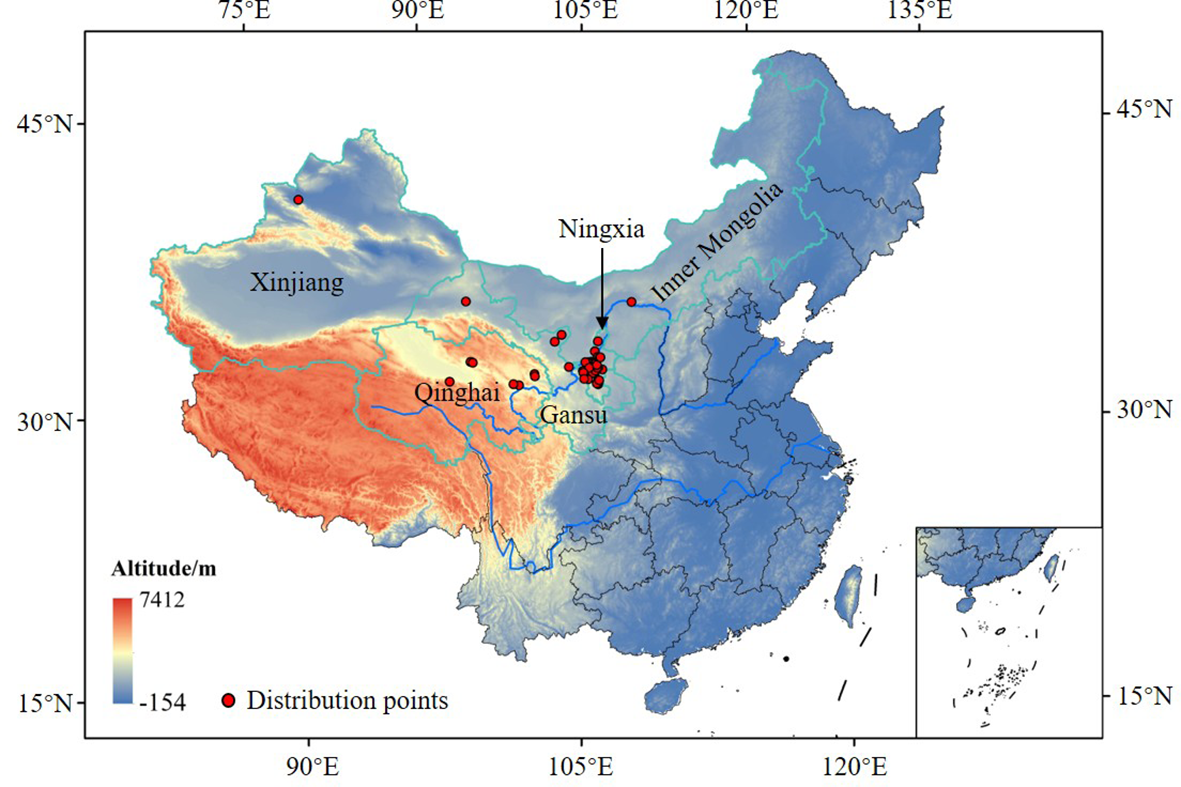
The filtered distribution points of *G. lycantha* in China.

In China, the primary goji berry production areas were concentrated in the Northwestern region (35°05′–42°50′ N, 92°13′–108°46′ E), at an altitude of approximately 1500 m, including Ningxia, Gansu, Qinghai, Xinjiang and Inner Mongolia (**Figure 2**). The study area has a temperate continental climate, with an annual average temperature of 3–10 °C and annual precipitation of 100–400 mm. Sufficient sunlight and large diurnal temperature differences are conducive to the synthesis and accumulation of active ingredients in goji berry (Song et al., 2025).

### 2.2 Distributed data acquisition and processing

The distribution points of the midge were primarily obtained from the Medicinal Plant Pests Database (https://www.pests.cn/index/tu?pic_name=%E6%9E%B8%E6%9D%9E%E7%BA%A2%E7%98%BF%E8%9A%8A, accessed 19 August 2024), published literature (Xu et al., 2014), and field investigations (2023–2024), with a total of 79 distribution points collated. To reduce the influence of sampling bias on prediction results, these distribution points were filtered using the buffer analysis method in ArcGIS 10.7 (https://www.esri.com/). Only one point was retained when the distance between two points was less than 10 km (Wu et al., 2025). Finally, 56 distribution points were retained for subsequent modelling (**Figure 2**, **Supplementary Table 1**). The geographical base map of China was from the Standard Map Service System of the Ministry of Natural Resources (http://bzdt.ch.mnr.gov.cn/download.html?searchText=GS%2520(2019)%25201822) [Inspection number: GS (2019) 1822].

### 2.3 Environmental data acquisition and processing

Nineteen climate factors and 17 soil factors were utilized to predict the current and future suitable habitats of the midge (**Table 1**). For consistency, the spatial resolution of the 36 environmental factors was 2.5 arc-minutes (5 km). Climate factors were obtained from the WorldClim database (WorldClim v2.1, https://www.worldclim.org/), covering the time period from 1970 to 2000. Future 2050s (averages for 2041–2060) and 2070s (averages for 2061–2080) climate data were selected from four Shared Socioeconomic Pathways (SSPs) under the Coupled Model Intercomparison Project Phase 6 (CMIP6), which had a good predictive effect in China. These included low greenhouse gas emission (SSP126), medium greenhouse gas emission (SSP245), high greenhouse gas emission (SSP370), and extremely high greenhouse gas emission (SSP585) (Eyring et al., 2016; Tan et al., 2022). Due to the habit of pupating in the soil of the midge, soil characteristics might influence its emergence and survival (Deng et al., 2025). Soil factors were obtained from the Harmonized World Soil Database (HWSD v1.2, http://www.fao.org/soils-portal/soil-survey/soil-maps-and-databases/harmonized-world-soil-database-v12/en/). Given the present lack of future soil data, this study assumed that soil conditions would remain unchanged over time (Li et al., 2024a; Song et al., 2024).

**Table 1:** Detailed information on the 36 environmental factors.

Factor	Description (Unit)	Factor	Description (Unit)
bio1	Annual mean temperature (°C)	bio19	Precipitation of coldest quarter (mm)
bio2	Mean diurnal temperature range (°C)	t_gravel	Volume percentage of gravel (%)
bio3	Isothermality	t_sand	Percentage of sand (%)
bio4	Temperature seasonality	t_silt	Percentage of silt (%)
bio5	Maximum temperature of warmest month (°C)	t_clay	Percentage of clay (%)
bio6	Minimum temperature of coldest month (°C)	t_usda_tex	Texture class name and code
bio7	Temperature annual range (°C)	t_ref_bulk_density	Cation exchange capacity (g/cm³)
bio8	Mean temperature of wettest quarter (°C)	t_oc	Percentage of organic carbon (%)
bio9	Mean temperature of driest quarter (°C)	t_ph_h2o	Soil reaction
bio10	Mean temperature of warmest quarter (°C)	t_cec_clay	Cation exchange capacity of the clay fraction (cmol/kg)
bio11	Mean temperature of coldest quarter (°C)	t_cec_soil	Cation exchange capacity (cmol/kg)
bio12	Annual precipitation (mm)	t_bs	Base saturation (%)
bio13	Precipitation of wettest month (mm)	t_teb	Total exchangeable bases (cmol/kg)
bio14	Precipitation of driest month (mm)	t_caco3	Calcium carbonate content (%)
bio15	Precipitation seasonality (mm)	t_caso4	Calcium sulfate content (%)
bio16	Precipitation of wettest quarter (mm)	t_esp	Exchangeable sodium percentage (%)
bio17	Precipitation of driest quarter (mm)	t_ece	Electrical conductivity (dS/m)
bio18	Precipitation of warmest quarter (mm)	t_texture	Topsoil texture

To reduce multicollinearity and improve the prediction accuracy of the MaxEnt model (Han et al., 2025), Pearson correlation analysis was conducted on 36 environmental factors using ENMTool (https://github.com/) (**Supplementary Figure 1**). The default parameter settings of the MaxEnt model v3.4.1 (https://biodiversityinformatics.amnh.org/open_source/maxent/) were adopted, and the environmental factors and distribution points were imported into the model, and the jackknife analysis was used to evaluate the contribution rates of the 36 environmental factors. The environmental factors with zero contribution were excluded. Factors with an absolute correlation coefficient greater than 0.8 were retained only when they reached the highest contribution in the jackknife analysis (Li et al., 2024a). Finally, 11 environmental factors for model construction and prediction were screened out.

### 2.4 Model parameter optimization

Since the feature combinations (FC) and regularization multiplier (RM) significantly influence the stability of the MaxEnt model, this study utilized the “kuenm” package (https://github.com/) in R v3.6.3 (https://www.r-project.org/) to optimize the two parameters. During the optimization process, a total of 1,240 parameter combinations were evaluated by combining 31 feature combinations (L, Q, H, P, and T) with regularization multipliers ranging from 0.1 to 4.0 (0.1 increments). 75% of the occurrence data were randomly selected as training data, and the remaining 25% as testing data. The parameters associated with the minimum values of both the omission rate and delta AICc were selected as the optimal model parameters (Deng et al., 2024).

The screened distribution data of the midge and environmental factors were imported into MaxEnt. The optimal parameter combination of FC and RM was selected, with 75% of the distribution points as training data and the remaining 25% as testing data. The jackknife method was applied to evaluate the contribution of key environmental factors. The output format was set to Logistic, and the repeated run type was Subsample, with the simulation repeated 10 times. All other parameters were set to default.

The area under the receiver operation characteristic curve (AUC) and true skill statistic (TSS) could be used to evaluate the accuracy of prediction results. The closer the AUC and TSS values were to 1.0, the higher the predictive accuracy. When the AUC and TSS values were greater than 0.90 and 0.75, respectively, the model was considered to exhibit excellent predictive performance (Shi et al., 2023; Song et al., 2025).

### 2.5 Classification and area calculation of suitable habitats

The MaxEnt prediction results were reclassified and visualized using ArcGIS. The Maximum Test Sensitivity Plus Specificity (MTSPS) threshold was employed as a standard criterion for defining the boundaries of species’ suitable habitats (Wang et al., 2025). According to this threshold, the suitable habitats of the midge were divided into unsuitability (0–MTSPS), low suitability (MTSPS–0.4), moderate suitability (0.4–0.6), and high suitability (0.6–1.0) (Li et al., 2024a).

The number of grids occupied by each suitable habitat and the total number of grids in China were counted. The area of each habitat in China and in the primary goji berry cultivation provinces (Ningxia, Gansu, Inner Mongolia, Xinjiang, and Qinghai) under current and future (2050s, 2070s) climate scenarios was calculated based on the proportion of their grids to China’s total land area (Song et al., 2024).

### 2.6 Spatiotemporal changes and centroid analysis

To display the changes in potential suitable habitats of the midge under current and future climate scenarios, the SDM Toolbox (v2.5, http://www.sdmtoolbox.org/) was utilized. Utilizing the “Distribution Changes Between Binary SDMs” function, and the values were defined as 0 for unsuitability, –1 for expansion, 1 for stable, and 2 for reduce, and the areas of these four types of changes were calculated using the method described in Section 2.5. Furthermore, the “Centroid Changes (Lines)” function was used to calculate the migration distance and location of the habitat centroids (Li et al., 2024b).

## 3 Results

### 3.1 Model optimization results and evaluation

**Figure 3:**
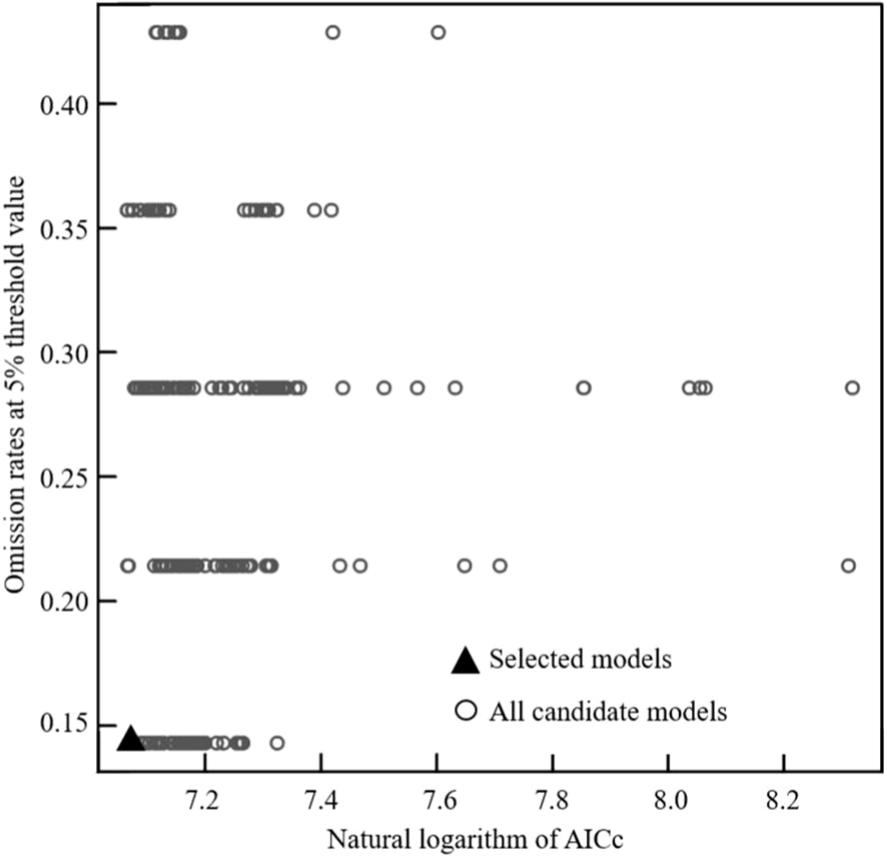
Optimal model parameter combination.

After optimization, the values of omission rate and delta AICc reached their minimum. Under this condition, the optimal parameters for the MaxEnt model were identified as FC = HP (Hinge + Product) and RM = 1 (**Figure 3**). Based on the optimized model parameter settings, the MaxEnt model prediction results showed a AUC value as high as 0.981 and a TSS value of 0.974, indicating that the model was accurate and could effectively predict the suitable habitats of the midge.

### 3.2 Dominant environmental factors influencing the distribution of *G. lycantha*

**Figure 4:**
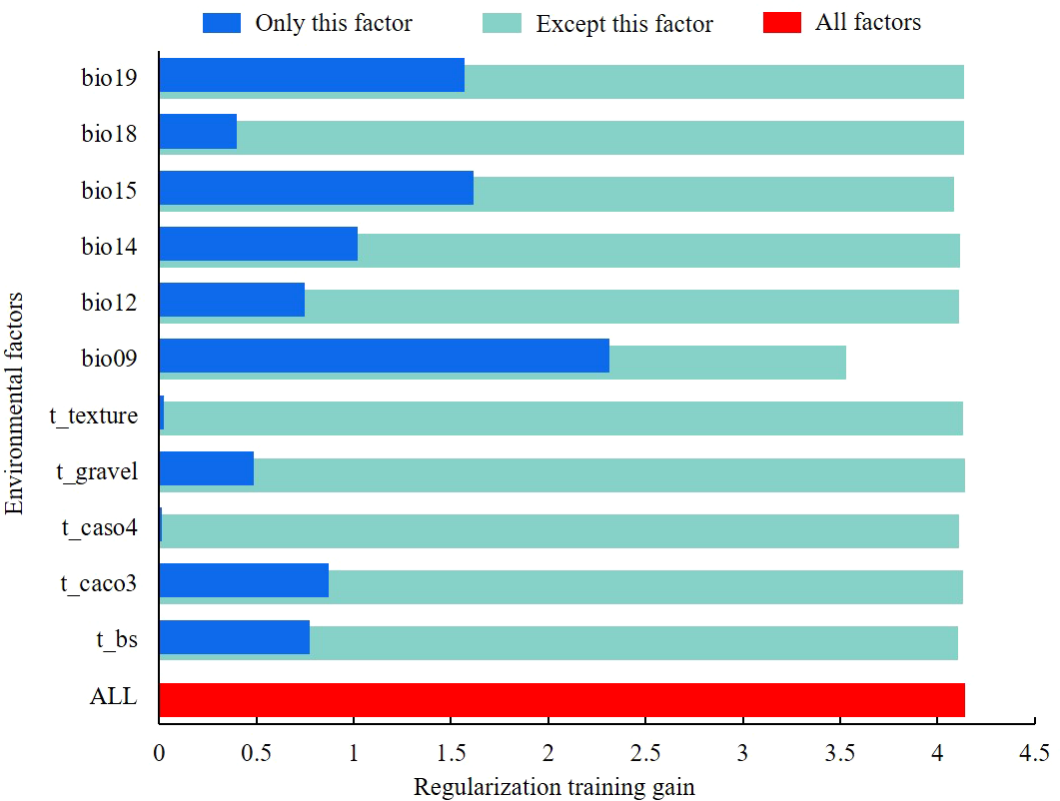
Jackknife test result of environmental factors.

The environmental factors influencing the distribution of the midge were comprehensively analyzed based on the jackknife of regularized training gain and the contribution rate. When only this factor was considered (**Figure 4**), the mean temperature of driest quarter (bio9) had the greatest impact on the regularized training gain, followed by precipitation seasonality (bio15) and precipitation of coldest quarter (bio19).

From the perspective of environmental factor contribution rates (**Table 2**), the environmental factors with the highest contribution rate to the model were mean temperature of driest quarter (bio9, 40.5%), precipitation of coldest quarter (bio19, 26.1%), and precipitation seasonality (bio15, 15.4%), with a cumulative contribution rate of 82.0%.

**Table 2:** Importance of environmental factors.

Factor	Contribution rate (%)
bio9	40.5
bio19	26.1
bio15	15.4
t_bs	5.5
bio12	4.9
bio18	3.1
bio14	2.5
t_caco3	1.4
t_caso4	0.5
t_gravel	0.1
t_texture	0.1

**Figure 5:**
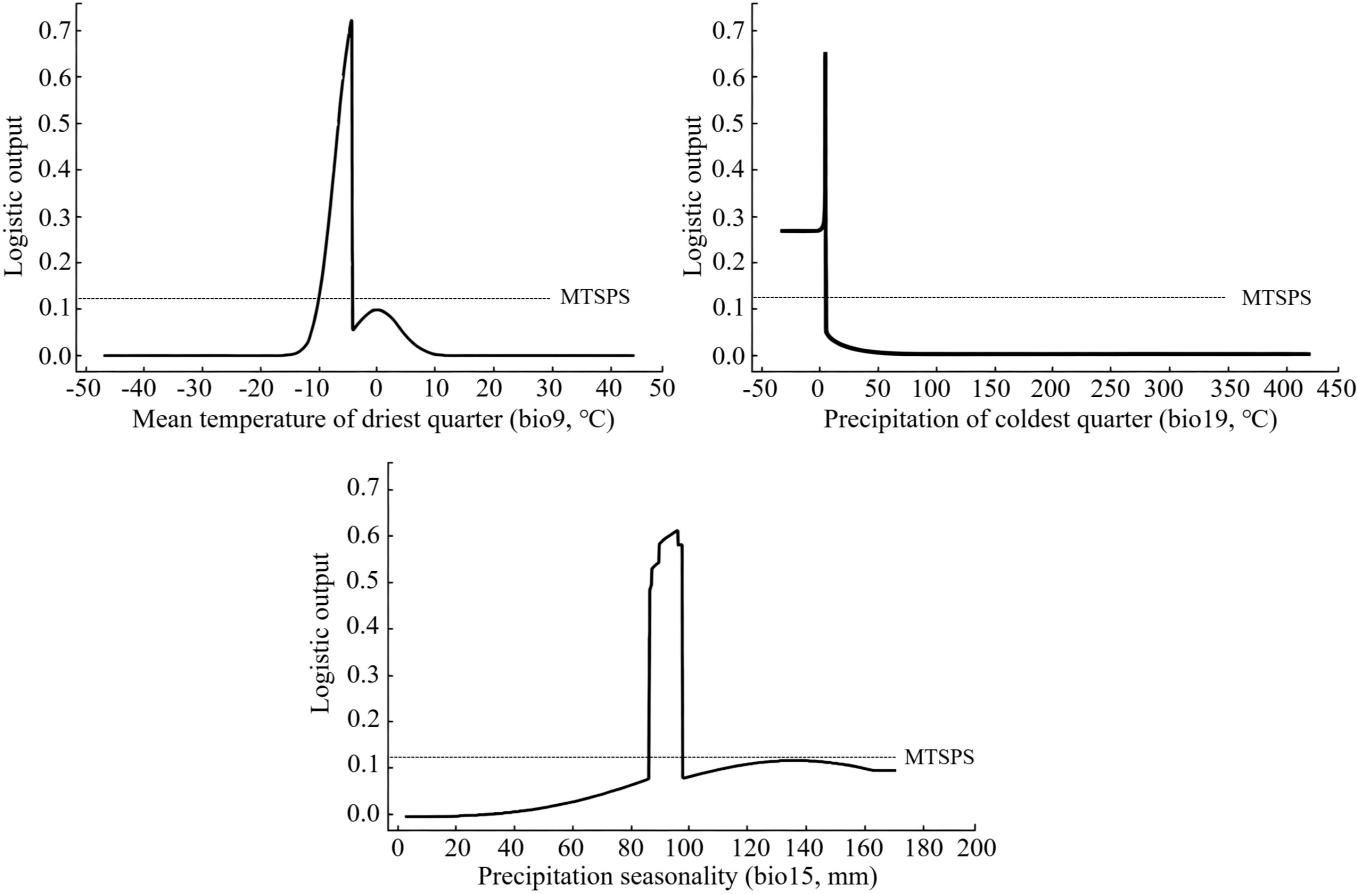
Response curve of dominant environmental factor.

To visualize the response curves of the main environmental factors, factors with a logistic output value exceeding 0.1693 (MTSPS) were selected, indicating that the corresponding environmental conditions were favorable for the growth and development of the midge. Under this threshold, the suitable response ranges of the key environmental factors were bio09 (-9.36–4.43 °C), bio19 (≤7.98 mm), and bio15 (87.85%–99.43%) (**Figure 5**, **Supplementary Figure 2**).

### 3.3 Suitable habitats under different climate scenarios

#### 3.3.1 Current suitable habitats

**Figure 6:**
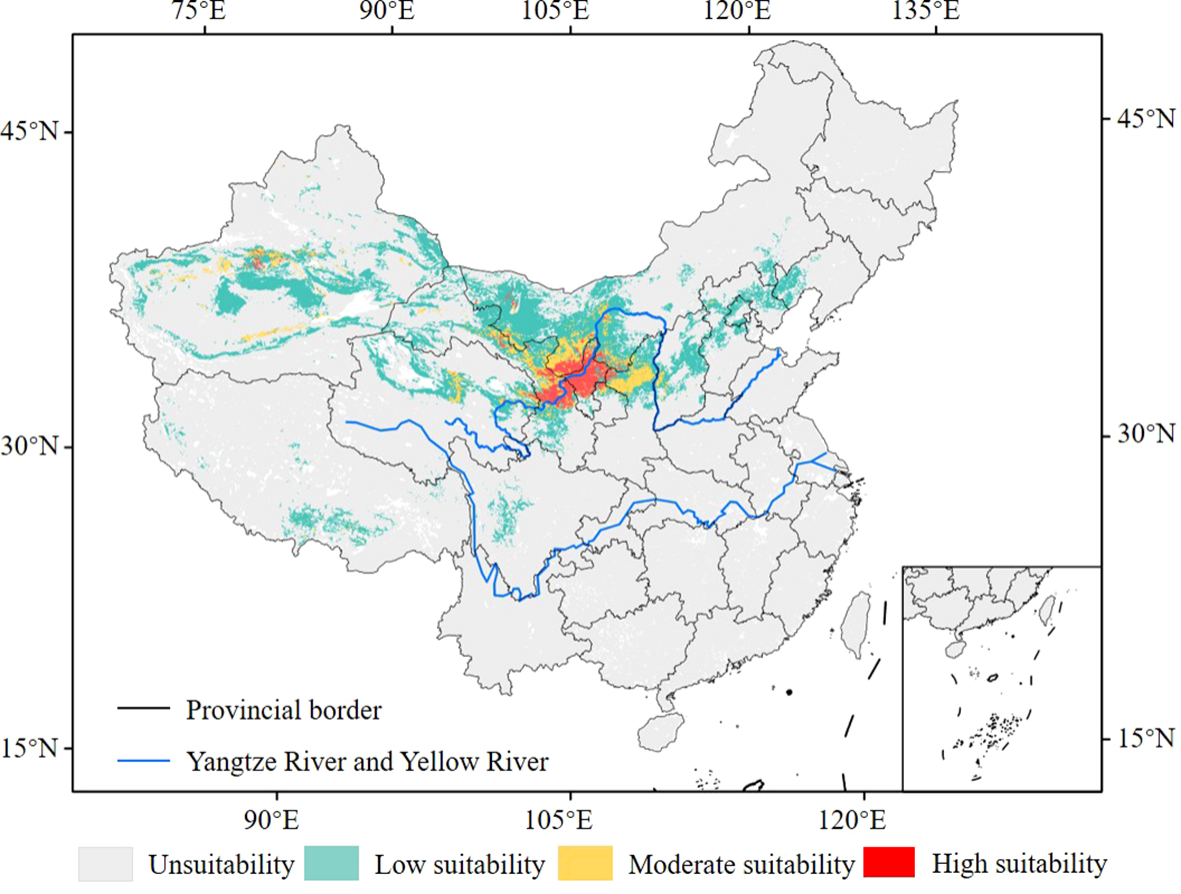
Distribution of suitable habitats of *G. lycantha* in China under current climate.

**Figure 7:**
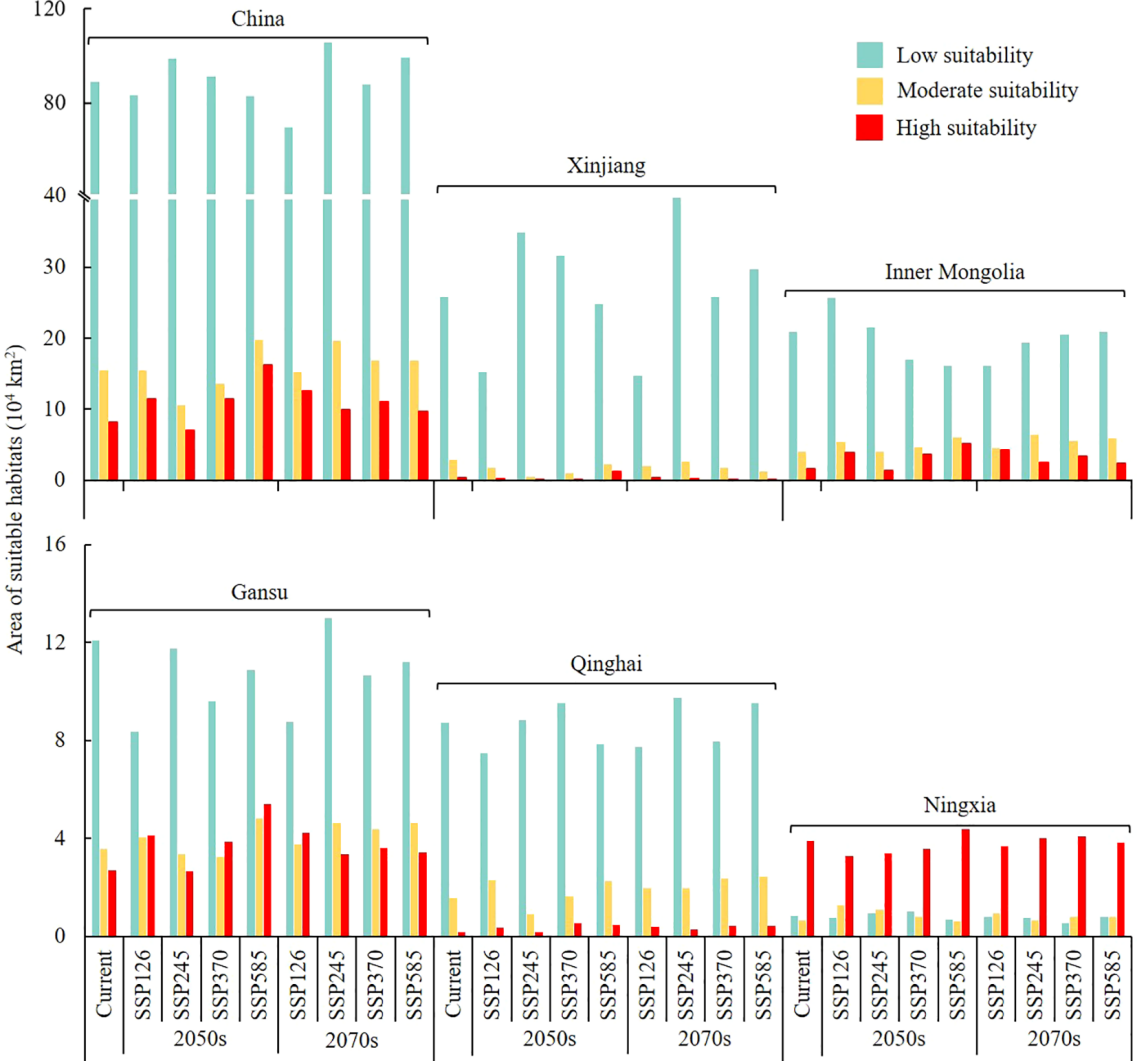
Area of the suitable habitats of *G. lycantha* under current and future climate scenarios.

The suitable habitats of the midge were mainly concentrated in northwestern China (**Figure 6**). The total area of the suitable habitats was approximately 112.73 × 10^4^ km^2^, accounting for 11.74% of China’s land area (**Figure 7**). The area of the highly suitable habitats was approximately 8.79 × 10^4^ km^2^, accounting for 7.80% of the total suitable habitat area. It was primarily distributed in Ningxia (3.90 × 10^4^ km^2^), central Gansu (2.71 × 10^4^ km^2^), southern Inner Mongolia (1.59 × 10^4^ km^2^), and scattered areas such as eastern Qinghai and central Xinjiang (0.59 × 10^4^ km^2^). The area of moderately suitable habitat was approximately 15.47 × 10^4^ km^2^, accounting for 13.72% of the total suitable habitat area. It was mainly distributed in the eastern and northwestern regions adjacent to the highly suitable habitats. In China, the suitable habitats for the midge were predominantly classified as low-suitability areas, covering approximately 88.47 × 10^4^ km^2^ and accounting for 9.22% of the country’s total land area. These areas were primarily distributed in North China and Northwest China.

#### 3.3.2 Future suitable habitats

Under different future (2050s and 2070s) climate scenarios, the distribution of the suitable habitats of the midge remained largely consistent with the current distribution, primarily concentrated in North China and Northwest China.

In the 2050s and 2070s, with increased greenhouse gas emissions, the area of suitable habitats for the midge under SSP245, SSP370, and SSP585 climate scenarios expanded, except for the SSP126 scenario. Among the four climate scenarios in the future, the suitable area under SSP585-2050s (119.06 × 10^4^ km^2^) and SSP245-2070s (135.25 × 10^4^ km^2^) reached their maximum, with an increase of 5.62% and 19.98% compared with the current area, respectively (**Figure 7**).

**Figure 8:**
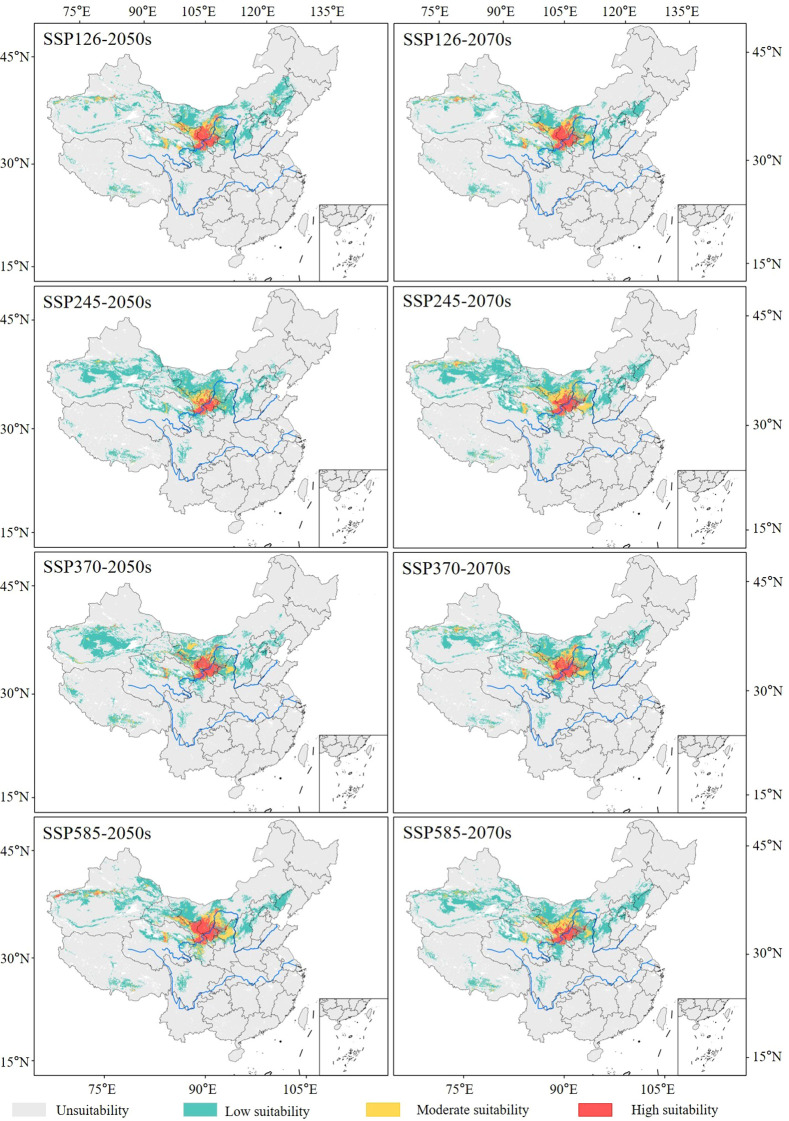
Distribution of suitable habitats of *G. lycantha* in China under different future climate scenarios.

Under future climate scenarios, Xinjiang was the province most severely affected by the midge, followed by Inner Mongolia, Gansu, Qinghai, and Ningxia (**Figure 8**). The area of suitable habitats in Xinjiang (42.64 × 10^4^ km^2^) and Gansu (20.94 × 10^4^ km^2^) reached their maximum under SSP245-2070s, with increases of 46.89% and 14.08%, respectively. In Inner Mongolia, the suitable area reached its maximum (34.98 × 10^4^ km^2^) under SSP126-2050s, increasing by 32.29%. In Qinghai, the suitable area reached its maximum (12.39 × 10^4^ km^2^) under SSP585-2070s, increasing by 18.38%. In Ningxia, the suitable area reached its maximum (5.67 × 10^4^ km^2^) under SSP585-2050s, increasing by 5.24%.

### 3.4 Spatiotemporal and centroid changes in the suitable habitats of *G. lycantha*

**Figure 9:**
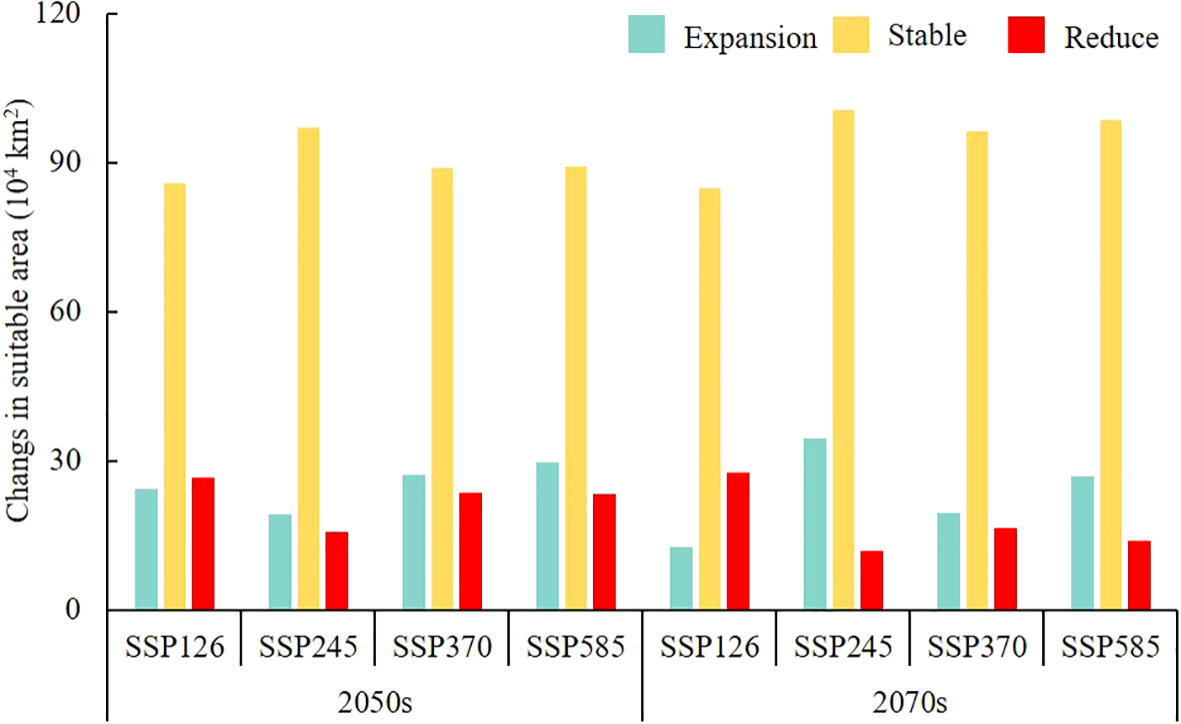
Spatiotemporal changes in the area of suitable habitats under different future climate scenarios compared with the current.

**Figure 10:**
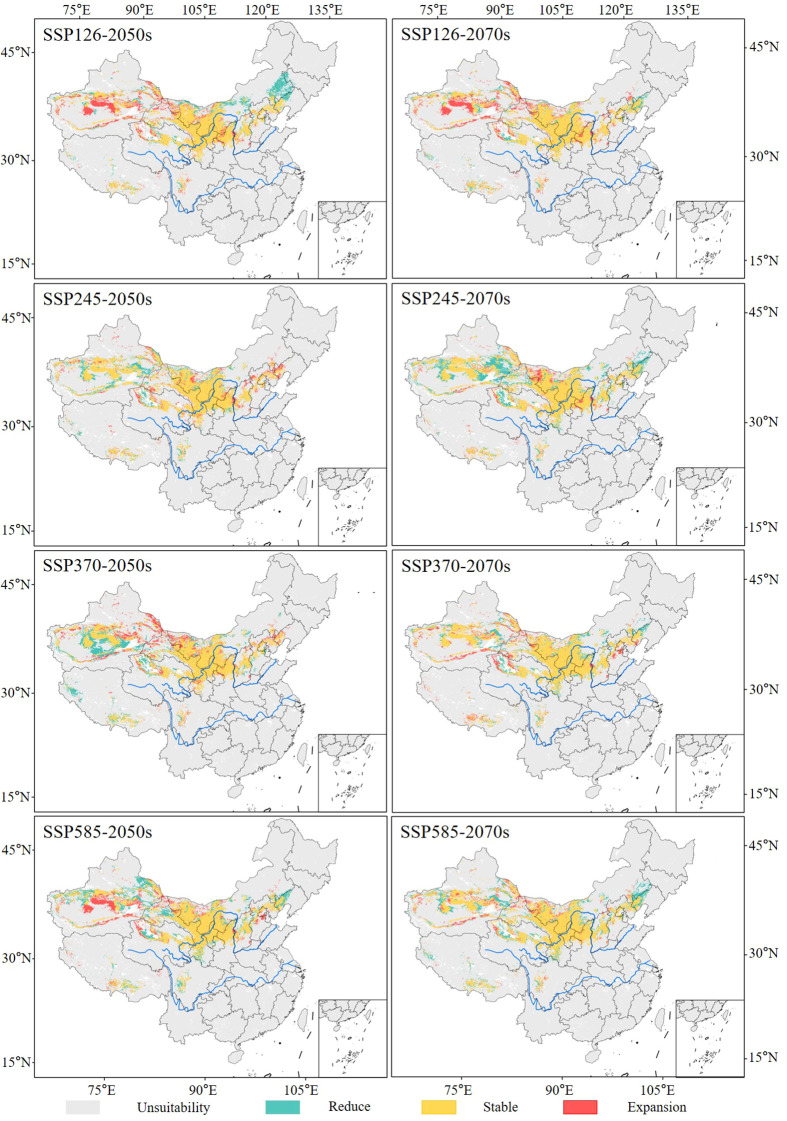
Dynamic changes in suitable habitats of *G. lycantha* under different climate scenarios.

Compared with the current climatic conditions, 76.26%–86.00% of suitable habitats remained stable in the 2050s, with the expansion area reaching a maximum of 29.77 × 10^4^ km^2^ in the SSP585 scenario. In the 2070s, 75.40%–89.37% of suitable habitats remained stable, with the expansion area reaching a maximum of 34.51 × 10^4^ km^2^ in the SSP245 scenario (**Figure 9**). Notably, the expansion regions were mainly concentrated in southern Xinjiang Province, Haixi Mongolian and Tibetan Autonomous Prefecture of Qinghai Province, and northwestern Gansu Province (**Figure 10**).

**Figure 11:**
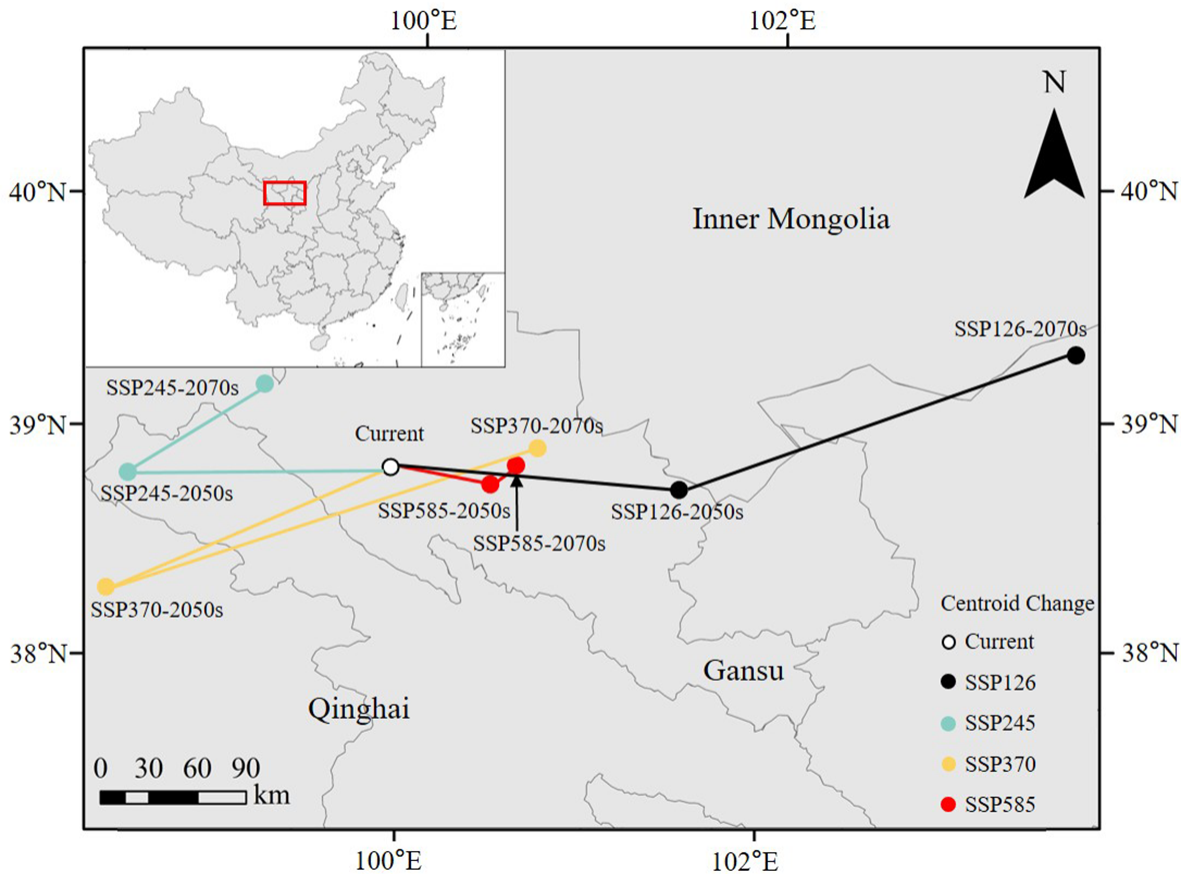
Changes in the centroid of suitable habitat of *G. lycantha* in China.

The movement of the centroid reflected variations in environmental factors influencing habitat suitability, and future changes in climate, soil, and other environmental conditions would influence the potential distribution of the midge. The centroid of the suitable habitats under current climate conditions was in Zhangye prefecture-level city (99.55° E, 38.91° N), Gansu Province, bordering Qinghai (**Figure 11**). Compared with the current situation, the centroid of the suitable habitat shifted eastward (under SSP126 and SSP585) and westward (under SSP245 and SSP370) in the 2050s, with a migration distance of 59.50–155.36 km. Compared with the 2050s, the centroid of suitable habitats shifted northeastward in the 2070s, with a migration distance of 17.45–178.96 km.

## 4 Discussion

### 4.1 Analysis of key environmental factors in geographic distribution of *G. lycantha*

Since the midge had the habit of pupating in soil, Deng et al. (2025) demonstrated that different soil factors significantly affected the emergence of adult the midge. The most favorable conditions for its emergence were soil moisture content of 15%, soil bulk density of 1.0 g/cm³, and soil depth of 5 cm. However, the results of this study indicated that the total contribution rate of soil factors to the distribution of the midge was 7.5%, while that of climatic factors was 92.5%. This discrepancy highlighted that while soil factors played a significant role in the pupation process of the midge, climate factors remained the primary driver of the species’ geographical distribution at the macro-scale. For insects, the rates of key physiological processes in their life cycles (development, eclosion, reproduction) were determined by environmental conditions, especially temperature and precipitation factors that render insect distributions highly sensitive to changes in climatic factors (Régnière, 2009). For instance, Li et al. (2025b) reported that climatic factors had a significantly higher contribution rate than soil factors in influencing the distribution of *L. decempunctata*, which was consistent with the results of this study.

The interaction among the environment and host plant directly affected the spatiotemporal distribution and dynamics of pests (Wang and Qin, 2007). According to the contribution rates and regularized training gain, the mean temperature of the driest quarter was identified as the dominant climatic factor affecting the distribution of the midge, with a suitable range of -9.36 to -4.43 °C. Although no studies had been reported on the minimum lethal temperature of the midge, combined with its biological characteristic of overwintering in soil, it could be inferred that excessively low temperatures during the overwintering period might prevent overwintering pupae from surviving due to cold stress. Conversely, unusually high winter temperatures might break the diapause of overwintering pupae, leading to premature development. However, the emerged adults would struggle to survive and reproduce as they lacked host flower buds for oviposition (Li et al., 2025b). Existing research showed that the mean temperature of the driest quarter was one of the key environmental factors affecting the distribution of its host plant, *L. barbarum* (Wang et al., 2023). When this temperature ranged from -7.4 to -3.3 °C, the suitability probability of *L. barbarum* exceeded 56% (Shen et al., 2023). Notably, the suitability range of the midge corresponded closely to that of its host plant. This synchronization ensured that the midge’s reproductive cycle aligned with critical growth stages of the host, thereby ensuring the stability of its population.

### 4.2 Potential distribution of *G. lycantha* under climate change

Climate change could profoundly affect species’ potential distribution patterns and biodiversity (Bellard et al., 2012). For example, plants adapted to new environmental conditions and coped with climate change by adjusting their geographical distribution (Wei et al., 2018). Similarly, pests faced pressures from climate change, leading to shifts in their suitable habitats. This study found that the suitable habitat area of the midge in China exhibited an overall expanding trend, indicating that climate change had a significant positive impact on its distribution. Under future climatic conditions, the hydrothermal conditions in high-latitude regions underwent significant changes. The environmental stress that originally restricted the distribution of the midge in high-latitude regions gradually diminished, thereby promoting the northeastward migration of its population. Li et al. (2025a) found that the suitable habitats of Russian box thorn, *Lycium ruthenicum*, gradually shifted northeastward, which was consistent with the results of this study. Furthermore, similar distribution dynamics had been extensively documented across various other pests. For instance, research indicated that the potential suitable habitat of the Japanese orange fly, *Bactrocera tsuneoni*s (Miyake), in China was continuously expanding northward, facilitated by the species’ rapid adaptive evolution to localized thermal extremes (Mao et al., 2024). The northward shifts observed in *Monolepta signata* Oliver (Liu et al., 2024) and *N. asiatica* (Song et al., 2024) further corroborate that climate change was transforming previously unsuitable regions into suitable habitats for pest colonization. These shared migration patterns across diverse agricultural pests further substantiated that climate change was the primary driver changing their geographical distributions, while also posing an increasingly severe threat to agricultural security in high-latitude regions. Under the low greenhouse gas emission scenario (SSP126), the suitable habitat area of the midge exhibited an overall reduction trend. However, under medium and high greenhouse gas emission scenarios (SSP245, SSP370 and SSP585), it showed a general expansion trend. We speculated that temperature rise caused by the accumulation of carbon dioxide emissions was conducive to the population growth of the midge, and mitigating carbon dioxide emissions could inhibit its population expansion to a certain extent.

### 4.3 Potential limitations

Although the MaxEnt simulation results employed in this study demonstrated high accuracy, the method itself possessed inherent limitations. The predicted results reflected the theoretical distribution of the midge under various future climate scenarios, whereas actual population dynamics might be constrained by modeling uncertainties and other confounding factors. This study used soil factors derived from the HWSD, with the assumption that these factors remained constant under both current and future climate scenarios. This assumption did not account for the fact that climate change-driven shifts in soil hydrothermal conditions (such as soil moisture fluctuations and intensified salinization) might affect the survival and development of pupae in the topsoil. Additionally, the geographical distribution of the midge was influenced not only by environmental factors (climate and soil) but also by other biotic and abiotic factors, including human activities, terrain differences, natural enemies, as well as its own adaptability and evolutionary capacity (Pearson and Dawson, 2003; Zhao et al., 2022). All these factors might lead to certain discrepancies between the model’s predicted results and the actual future geographical distribution of the midge. Future work should integrate these elements to better predict habitat availability in a changing environment.

### 4.4 Control strategy

In practice, prevention and control zones can be delineated based on the simulation results, and targeted precision control measures can be implemented accordingly. For the highly suitable habitats of the midge in China, such as Xinjiang, Inner Mongolia, Gansu, Qinghai, and Ningxia, stricter control measures (deep plowing to kill pupae, physical trapping and spraying of pesticides) should be adopted to reduce the degree of damage to the host plant (Li et al., 2015). On the other hand, as the suitable habitats of the midge expand northeastward, regions such as Xinjiang, Qinghai, and Liaoning should strengthen the dynamic monitoring of the midge, and formulate effective monitoring and control strategies to prevent widespread outbreaks.

## 5 Conclusion

This study systematically predicted and analyzed the potential distribution of the midge, *G. lycantha*, in China under current and future climate scenarios using an optimized MaxEnt model. According to the prediction results, the suitable habitats of the midge were predominantly located in Northwest China, covering the provinces of Xinjiang, Inner Mongolia, Gansu, Qinghai, and Ningxia, with the mean temperature of the driest quarter being the dominant environmental factor limiting its distribution. Under future climate scenarios, the suitable habitats of the midge were projected to expand further (except for the SSP126), with a distribution centroid shifting toward the northeast. This study provides a scientific basis for the standardized management and site selection of goji berry cultivation, and offers data support for stakeholders to develop targeted pest control strategies, thereby mitigating the impact of the midge on the sustainable development of the goji berry industry. Furthermore, future studies should integrate multiple biotic and abiotic factors, and combine population genetics with ecological niche modeling to improve the accuracy of predictions regarding the midge’s distribution.

## Data Availability

The raw data supporting the conclusions of this article will be made available by the authors, without undue reservation.
