# Supplementary figures and images for: Predicting the expansion of Gephyraulus lycantha as a key pest of goji berry in China under climate change
Source: Front Plant Sci. 2026 Apr 10;17:1786710. doi: 10.3389/fpls.2026.1786710 (PMC13106313; doi:10.3389/fpls.2026.1786710)

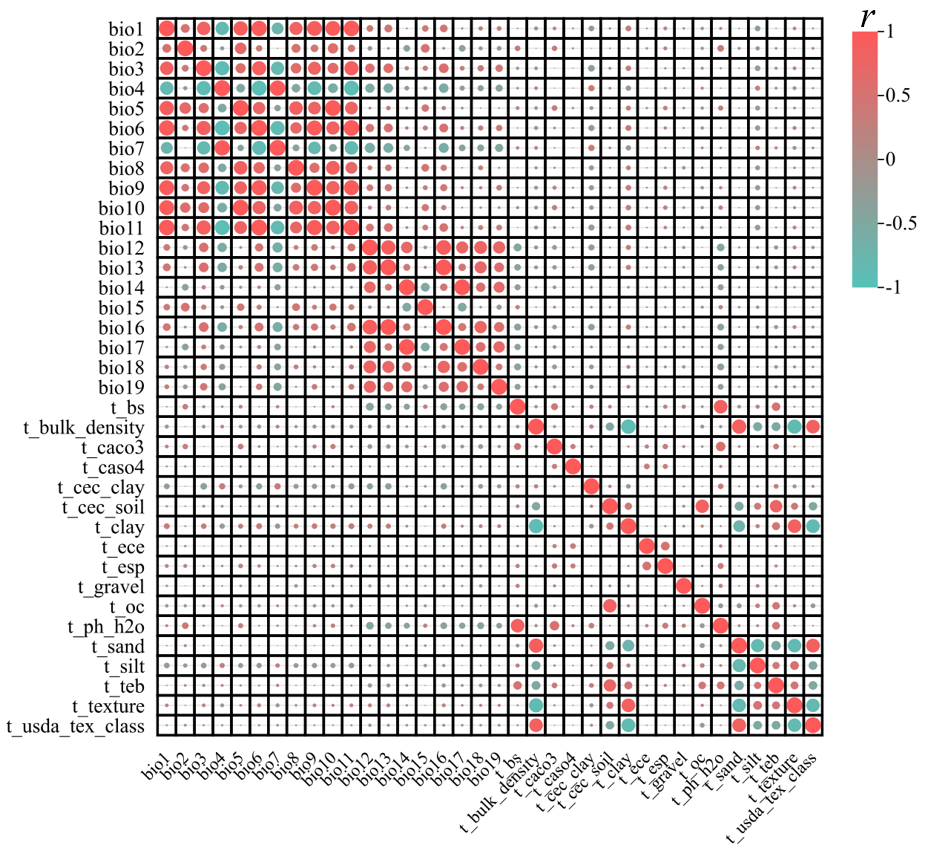

Supplement: Supplementary file 1 [file DataSheet1.zip › Supplementary Materials/Figure S1.png]

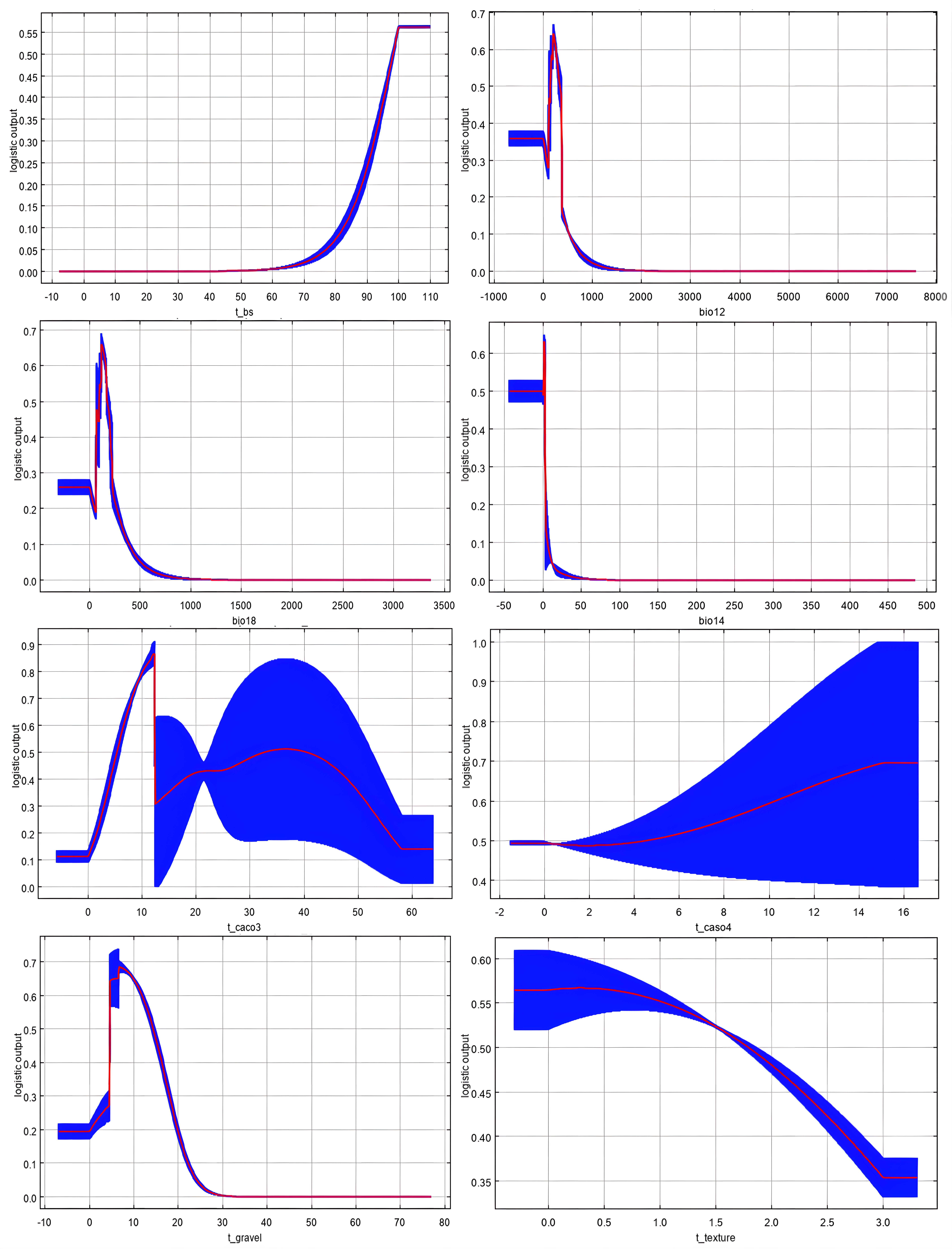

Supplement: Supplementary file 1 [file DataSheet1.zip › Supplementary Materials/Figure S2.png]
